# Supplementary material for: Exploring the proprioceptive potential of joint receptors using a biomimetic robotic joint
Source: Sci Rep. 2026 Feb 3;16:4724. doi: 10.1038/s41598-025-27311-3 (PMC12868901; doi:10.1038/s41598-025-27311-3)
Supplement: Supplementary file 1 — Supplementary Information. [file 41598_2025_27311_MOESM1_ESM.pdf]

Supplementary Information

Supplementary Figures

Supplementary Methods: Feature Analysis Methods of Deep Learning Models

Several methods have been proposed to quantitatively evaluate the importance of features in machine learning models, including Permutation Importance[1–3], SHAP values[4], and LIME[5]. Permutation Importance quantifies the importance of each feature by randomly shuffling it and measuring the resulting decrease in model performance. SHAP values, based on optimal transport theory, can capture interactions between features, but this method is computationally intensive. LIME, on the other hand, is specifically designed for explaining local predictions. In the redundancy analysis presented in the Analysis of Redundancy in Joint Receptors section, features were systematically reduced to examine the overall behavior of the model, requiring multiple iterations for each feature. To minimize computational costs while obtaining an overview of feature importance, Permutation Importance was chosen as the evaluation criterion. For the feature analysis in the Distribution of Critical Sensory Receptors Near Range of Motion Limits section, SHAP values were used despite their higher computational cost, as they account for dependencies between features from each joint receptor. In the Distribution of Critical Sensory Receptors Near Range of Motion Limits section, the model was trained five times for each of the twisting, bending, and push-pull movements, and the SHAP values were calculated. The results are presented in Fig. S5 for the twisting movement, Fig. S6 for the bending movement, and Fig. S7 for the push-pull movement.

Table 1: Table of Statistics on Positional Errors in Different Datasets.

Error Type	x (m)	y (m)	z (m)
Maximum Error	0.0209	0.0230	0.0276
Mean Error	0.0021	0.0023	0.0031
Standard Deviation	0.0033	0.0035	0.0047

Table 2: Table of Statistics on Attitude Errors in Different Datasets.

Error Type	roll (°)	pitch (°)	yaw (°)
Maximum Error	7.9132	5.3688	8.6223
Mean Error	0.3172	0.6780	1.2479
Standard Deviation	0.7047	0.8202	1.6711

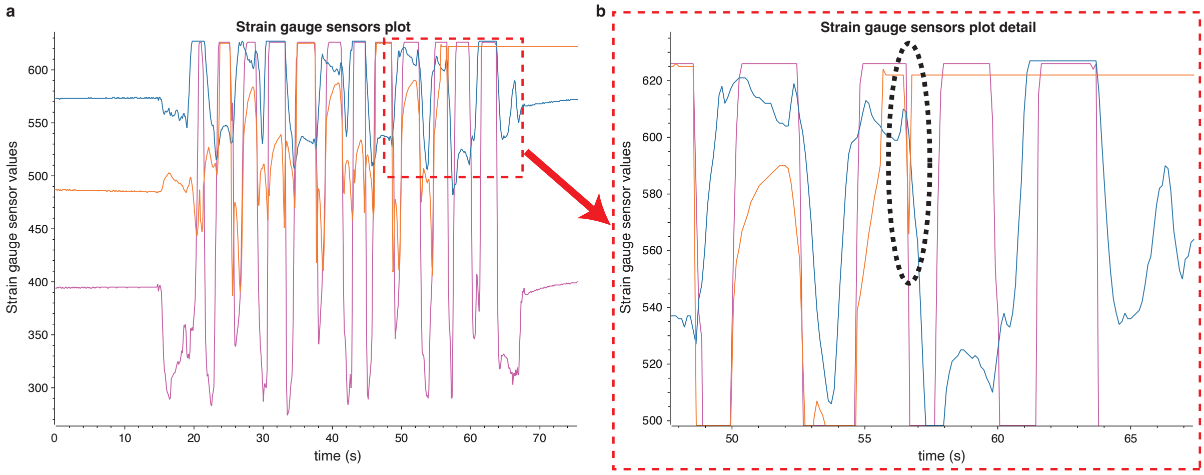

Supplementary Figure S1: Identification of disconnected strain gauge sensors. **a,** Plot of strain gauge sensors, including those with disconnections. For clarity, a selected subset of strain gauge sensors is plotted. **b,** Enlarged view of a portion of (a). The strain gauge sensor highlighted in orange within the dashed region is observed to be disconnected.

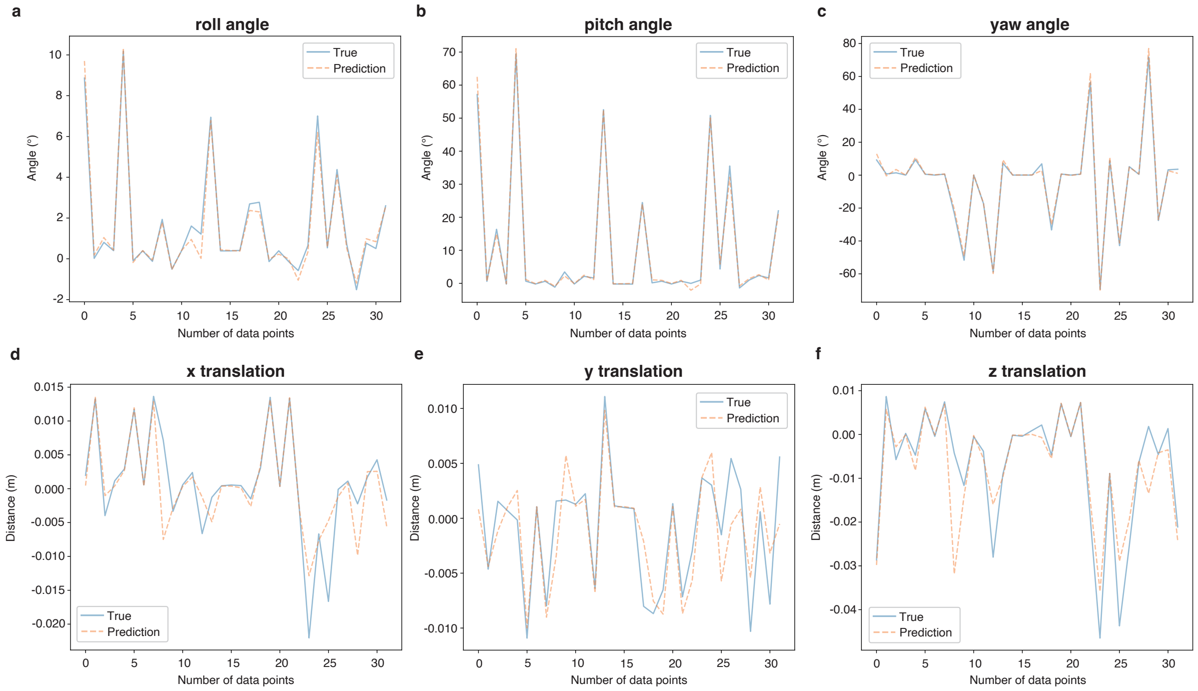

Supplementary Figure S2: Validation of deep learning–based proprioception estimation with an additional dataset. This figure shows the performance of the trained model when tested on a dataset collected independently but with the same procedure as in the Performance Verification of Joint Receptors Using Deep Learning section. The biomimetic joint is an open-type ball joint loosely connected by soft tissues, which permits not only orientations but also translational movements. The predicted values are compared against actual measurements (ground truth). **a-f,** This graph illustrates the joint coordinates and orientation estimated using deep learning based on sensory receptor data obtained from 60 strain gauge sensors embedded in the joint capsule. The model was trained in the same manner as in the Performance Verification of Joint Receptors Using Deep Learning section but using a different dataset collected through the same method. Due to the open-type ball joint being loosely connected by soft tissues, the graph visualizes not only orientation(roll (a), pitch (b), and yaw (c)) but also translation(x (d), y (e), and z (f) positions), comparing them to actual measurements (ground truth). The vertical axis represents angles for roll, pitch, and yaw, as well as position in meters, while the horizontal axis represents a dimensionless number denoting the number of test data points. The results indicate a high prediction accuracy.

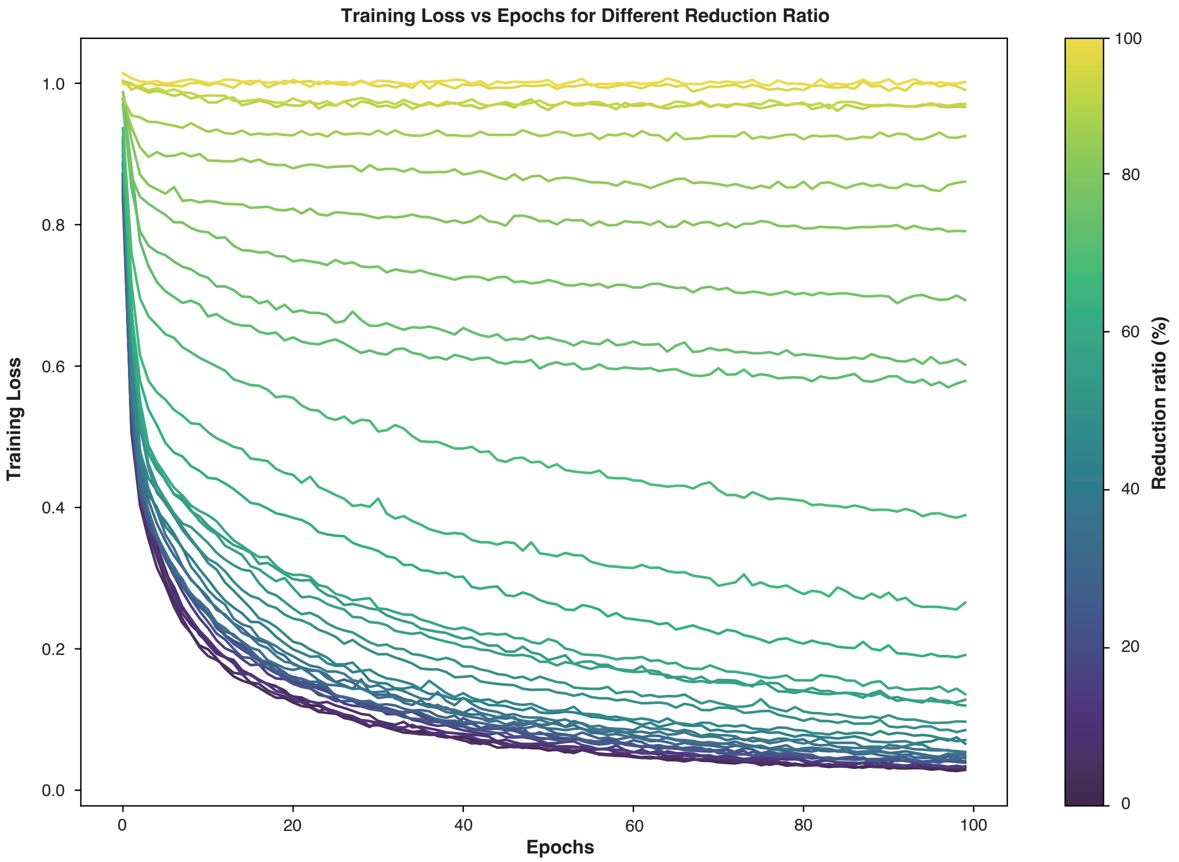

Supplementary Figure S3: Training process for redundancy analysis. This graph illustrates the training process used to examine the redundancy of proprioception acquired through joint receptors, as discussed in the Analysis of Redundancy in Joint Receptors section. The horizontal axis represents the number of training iterations (epochs), while the vertical axis represents the loss function. The reduction ratio is represented by a continuous color gradient, as indicated by the color bar. As the reduction ratio increases, the training process exhibits slower convergence and higher final loss values.

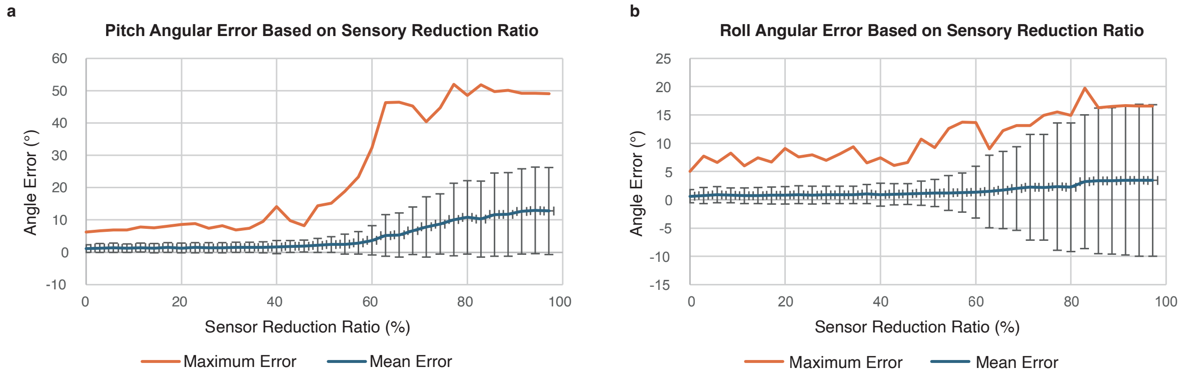

Supplementary Figure S4: Redundancy analysis of proprioception estimation under reduced sensor input from a single trial. This figure illustrates the angular errors for pitch (a) and roll (b) in one trial. Error bars represent the within-trial standard deviation of errors without scaling. Because this is a single trial, the results are subject to stochastic variation inherent in deep learning, and no statistical analysis can be performed. Nonetheless, visual inspection suggests a noticeable degradation in performance around a reduction ratio of approximately 50 %, which is consistent with the averaged results presented in the redundancy analysis of joint receptors in the main manuscript.

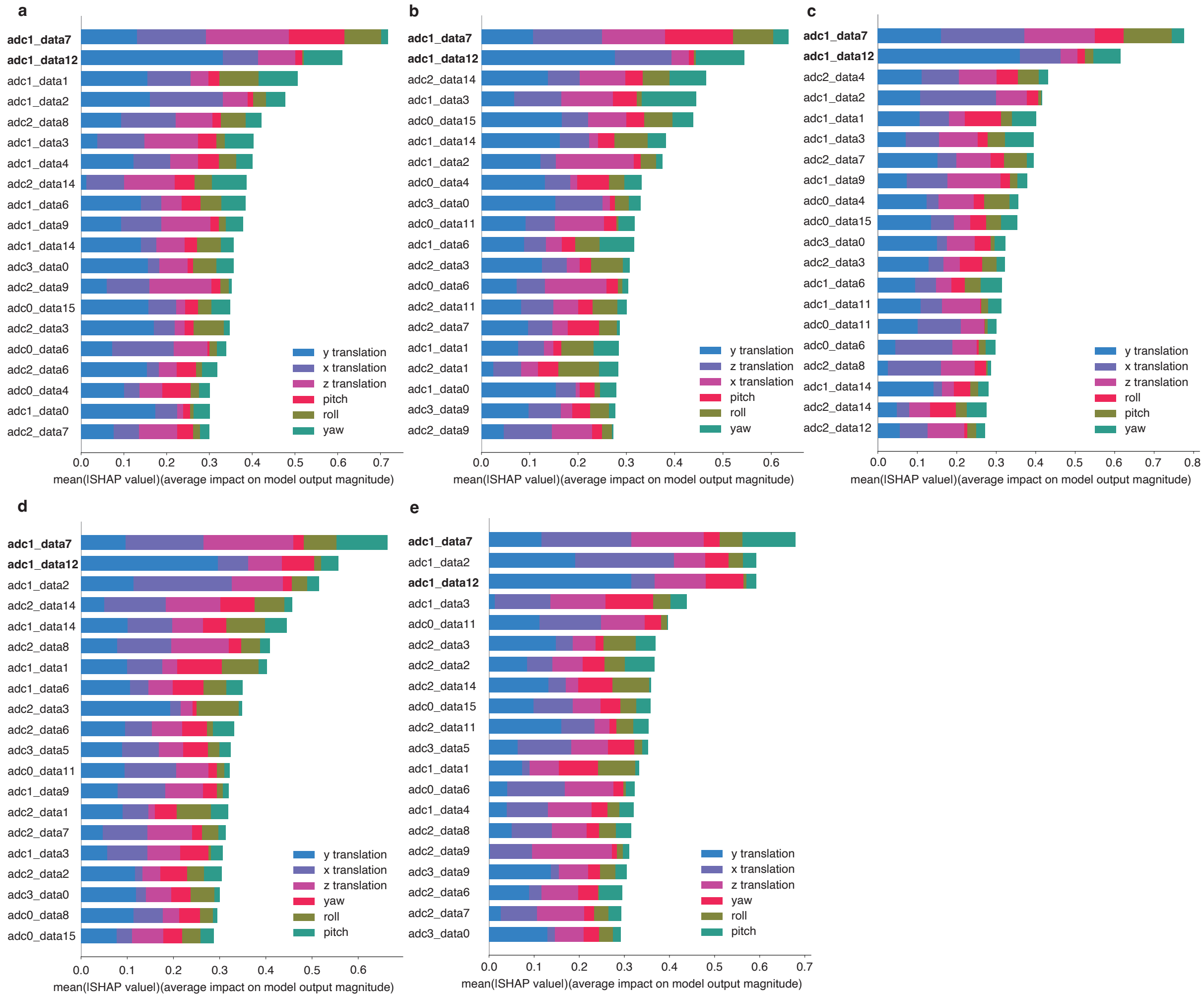

Supplementary Figure S5: Distribution of critical joint receptors in near-limit regions where twisting(roll) angles exceeded 20°. **a-e,** Bar graphs showing the results of five trials conducted in regions where twisting(roll) angles exceeded 20° in the Distribution of Critical Sensory Receptors Near Range of Motion Limits section. For each trial, SHAP values representing the contributions of all motion components (x, y, z, roll, pitch, and yaw) were aggregated to evaluate the overall importance of each sensory receptor. Among the top three most important receptors in each trial, 3, 6, and 6 were located in the mid-capsular, transitional, and bone attachment regions, respectively, indicating that receptors situated at or near the bone attachment were predominant. Notably, adc1_data7 and adc1_data12 were consistently included among the top three across all trials. These receptors are located in the bone attachment and transitional regions, respectively.

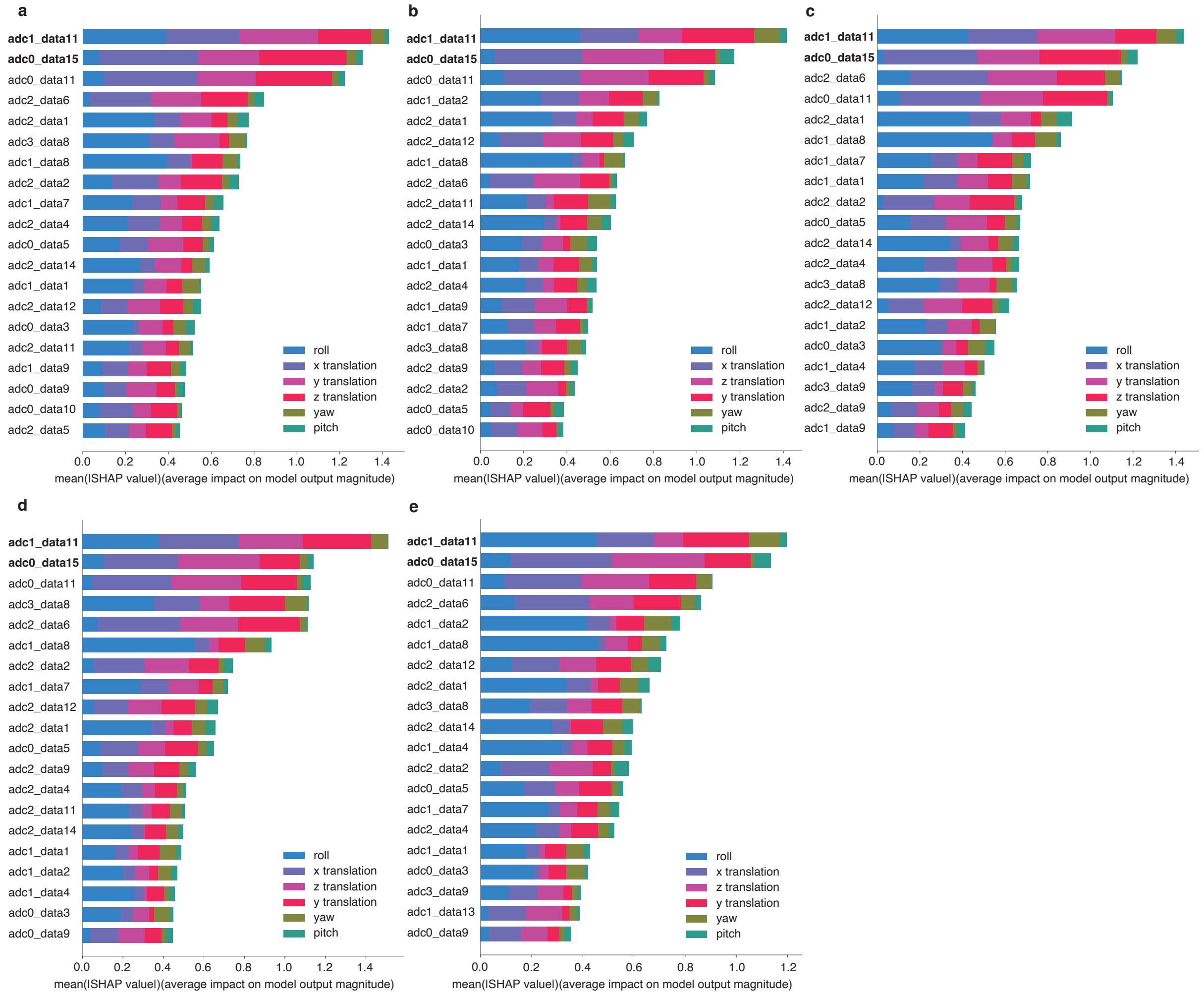

Supplementary Figure S6: Distribution of critical joint receptors in near-limit regions where bending(pitch or yaw) angles exceeded 70°. **a–e,** Bar graphs showing the results of five trials conducted in regions where bending(pitch or yaw) angles exceeded 70° in the Distribution of Critical Sensory Receptors Near Range of Motion Limits section. For each trial, SHAP values representing the contributions of all motion components (x, y, z, roll, pitch, and yaw) were aggregated to evaluate the overall importance of each sensory receptor. Among the top three most important receptors in each trial, 0, 9, and 6 were located in the mid-capsular, transitional, and bone attachment regions, respectively, indicating that receptors situated at or near the bone attachment were predominant. Notably, adc0_data15 and adc1_data11 were consistently included among the top three across all trials. These receptors are located in the bone attachment and transitional regions, respectively.

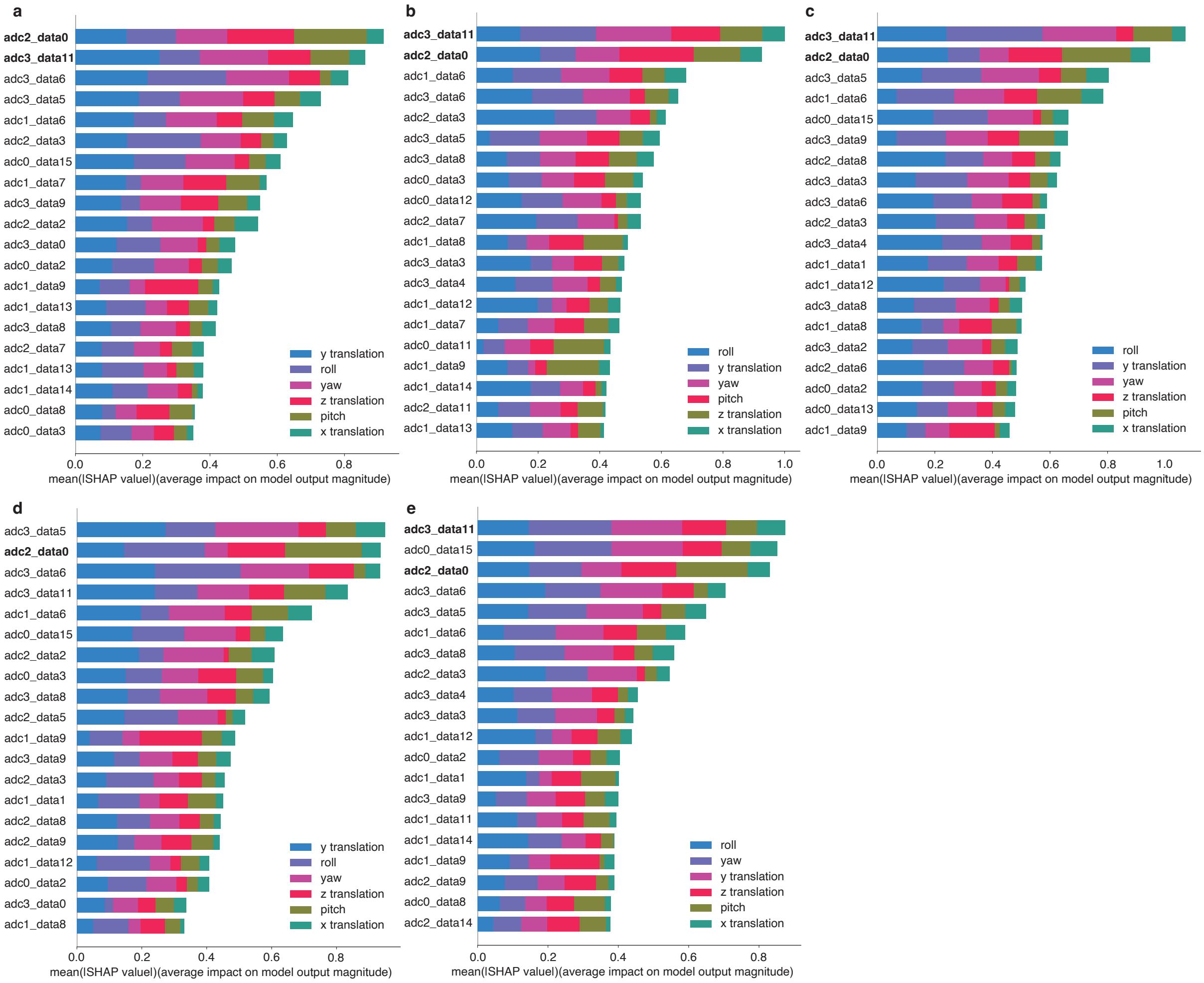

Supplementary Figure S7: Distribution of critical joint receptors in near-limit regions where the absolute value of x-translation exceeded 2 mm. **a–e,** Bar graphs showing the results of five trials conducted in regions where the absolute value of x-translation exceeded 2 mm in the Distribution of Critical Sensory Receptors Near Range of Motion Limits section. For each trial, SHAP values representing the contributions of all motion components (x, y, z, roll, pitch, and yaw) were aggregated to evaluate the overall importance of each sensory receptor. Among the top three most important receptors in each trial, 5, 6, and 4 were located in the mid-capsular, transitional, and bone attachment regions, respectively, indicating that mid-capsular receptors were also frequently identified as critical. Notably, adc3_data11 (transitional region) and adc2_data0 (mid-capsular region) were frequently included among the top three across the trials.

Supplementary References

References

[1] Leo Breiman. Random forests. *Machine learning*, 45:5–32, 2001.

[2] André Altmann, Laura Toloşi, Oliver Sander, and Thomas Lengauer. Permutation importance: a corrected feature importance measure. *Bioinformatics*, 26(10):1340–1347, 2010.

[3] Aaron Fisher, Cynthia Rudin, and Francesca Dominici. All models are wrong, but many are useful: Learning a variable's importance by studying an entire class of prediction models simultaneously. *Journal of Machine Learning Research*, 20(177):1–81, 2019.

[4] Scott M Lundberg and Su-In Lee. A unified approach to interpreting model predictions. In *Advances in Neural Information Processing Systems*, volume 30, pages 4765–4774, 2017.

[5] Marco Tulio Ribeiro, Sameer Singh, and Carlos Guestrin. " why should i trust you?" explaining the predictions of any classifier. In *Proceedings of the 22nd ACM SIGKDD international conference on knowledge discovery and data mining*, pages 1135–1144, 2016.
